# Recent Progress of Ion-Modified TiO_2_ for Enhanced Photocatalytic Hydrogen Production

**DOI:** 10.3390/molecules29102347

**Published:** 2024-05-16

**Authors:** Dongqiu Zhao, Xiao Tang, Penglan Liu, Qiao Huang, Tingxian Li, Lin Ju

**Affiliations:** 1 School of Physics and Electric Engineering, Anyang Normal University, Anyang 455000, China; dqzhao@aynu.edu.cn (D.Z.); 211104007@stu.aynu.edu.cn (Q.H.); wxlltx@126.com (T.L.); 2 Institute of Materials Physics and Chemistry, College of Science, Nanjing Forestry University, Nanjing 210037, China; xiaotang@njfu.edu.cn; 3 School of Science and Technology, Beijing Normal University•Hong Kong Baptist University United International College, Zhuhai 519087, China; lpl010507@163.com

**Keywords:** TiO_2_, photocatalysts, water splitting, dopants, hydrogen evolution reaction

## Abstract

Harnessing solar energy to produce hydrogen through semiconductor-mediated photocatalytic water splitting is a promising avenue to address the challenges of energy scarcity and environmental degradation. Ever since Fujishima and Honda’s groundbreaking work in photocatalytic water splitting, titanium dioxide (TiO_2_) has garnered significant interest as a semiconductor photocatalyst, prized for its non-toxicity, affordability, superior photocatalytic activity, and robust chemical stability. Nonetheless, the efficacy of solar energy conversion is hampered by TiO_2_’s wide bandgap and the swift recombination of photogenerated carriers. In pursuit of enhancing TiO_2_’s photocatalytic prowess, a panoply of modification techniques has been explored over recent years. This work provides an extensive review of the strategies employed to augment TiO_2_’s performance in photocatalytic hydrogen production, with a special emphasis on foreign dopant incorporation. Firstly, we delve into metal doping as a key tactic to boost TiO_2_’s capacity for efficient hydrogen generation via water splitting. We elaborate on the premise that metal doping introduces discrete energy states within TiO_2_’s bandgap, thereby elevating its visible light photocatalytic activity. Following that, we evaluate the role of metal nanoparticles in modifying TiO_2_, hailed as one of the most effective strategies. Metal nanoparticles, serving as both photosensitizers and co-catalysts, display a pronounced affinity for visible light absorption and enhance the segregation and conveyance of photogenerated charge carriers, leading to remarkable photocatalytic outcomes. Furthermore, we consolidate perspectives on the nonmetal doping of TiO_2_, which tailors the material to harness visible light more efficiently and bolsters the separation and transfer of photogenerated carriers. The incorporation of various anions is summarized for their potential to propel TiO_2_’s photocatalytic capabilities. This review aspires to compile contemporary insights on ion-doped TiO_2_, propelling the efficacy of photocatalytic hydrogen evolution and anticipating forthcoming advancements. Our work aims to furnish an informative scaffold for crafting advanced TiO_2_-based photocatalysts tailored for water-splitting applications.

## 1. Introduction

The rapid use of fossil fuels in recent decades has led to issues of energy scarcity and environmental degradation [1,2,3,4,5,6]. The urgency of finding secure, sustainable, green, and renewable sources of energy is particularly important, and various fossil fuel alternatives to current sources have emerged. Fortunately, solar energy, as a clean and inexhaustible natural resource, has been regarded as a promising sustainable energy source. However, solar energy cannot meet the needs of industries due to its naturally low energy flow density. The chemical conversion of solar photochemical storage and preservation is considered one of the most promising ways to use solar energy [5,7,8,9,10,11,12,13,14]. Using photocatalysts to produce hydrogen from water splitting with sunlight is anticipated to enable energy storage, addressing the present energy crisis and environmental issues [15,16]. Hydrogen energy is a clean and high-density energy source, and the product of its combustion, water, is also not a pollutant. Since the pioneering work on photoelectrocatalytic hydrogen production from water splitting by Fujishima and Honda in 1972 [17], there has been a surge of research on photocatalytic hydrogen production through the decomposition of water using semiconductor materials as catalysts [5,15,18,19]. Among all the semiconductors, TiO_2_ has received much attention due to its strong photocatalytic activity, availability, low cost, chemical stability, and nontoxicity [20,21]. However, TiO_2_ has a very low efficiency for photocatalytic hydrogen production from water splitting under sunlight. One reason is that the large bandgap (E_g_) of TiO_2_ (3.2 eV for anatase and 3.0 eV for rutile) can only be active to the ultraviolet (UV) light, which accounts for only about 4% of the total energy of sunlight, while visible (VIS) light, which accounts for about 45% of sunlight, is ineffective [22]. Another important factor is that the photogenerated electron–hole pairs of TiO_2_ are easily recombined [22,23]. In order to improve the utilization of sunlight and the separation and transfer of photoexcited carriers, researchers have tried various methods to modify TiO_2_ in recent years. In addition to morphological engineering through the design and fabrication of diverse nanostructures such as nanotubes [24], quantum dots [25], ultrathin nanosheets [26], and nanowires [27], to boost specific surface area, foreign dopant incorporation [28,29,30,31], such metal doping, metal nanoparticles (NPs) depositing, and nonmetal doping, has been demonstrated as an effective method to optimize the electronic property of TiO_2_ and expand the range of optical response.

Up to now, the research enthusiasm for ion-modified TiO_2_ photocatalysts has been flourishing. In order to provide basic knowledge to beginners and a detailed understanding to experts in the field, it is necessary to provide a systematic and comprehensive overview of the research progress on ion-modified TiO_2_ photocatalysts for hydrogen production. Most of the current reviews focused on the preparation methods and specific photocatalytic reactions. This paper reviews the recent progress of various modification strategies carried out on ion-modified TiO_2_ photocatalysts for hydrogen production from water splitting, containing the details of the modification methods and the effects of these methods on the efficiency of photocatalytic hydrogen production. Finally, we offer insights into ion-modified TiO_2_ photocatalysts, potentially advancing the creation of highly efficient photocatalysts for hydrogen generation through water splitting. 

## 2. Principle of Photocatalytic Water Splitting through Semiconductor

**Figure 1:**
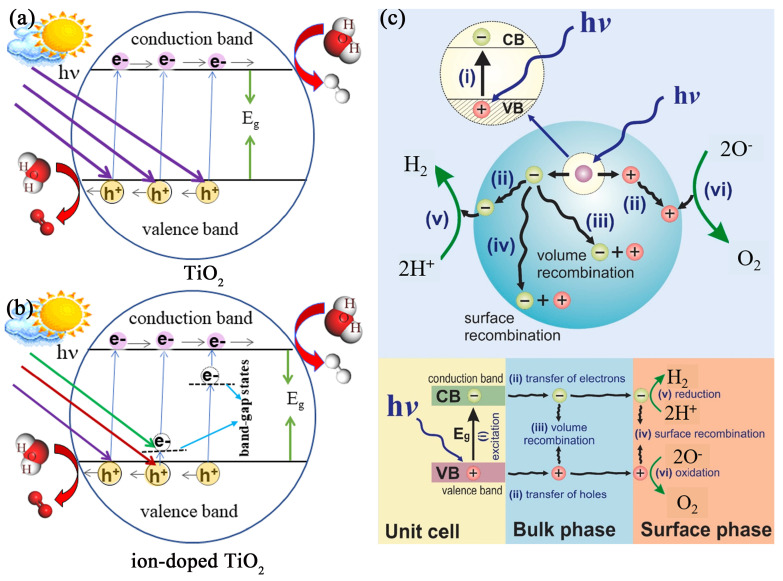
Diagram illustrating the process of hydrogen generation through photocatalytic water splitting: (**a**) for TiO_2_ and (**b**) for ion-doped TiO_2_. (**c**) Schematic representation of fundamental photocatalytic reactions in TiO_2_ [28].

Figure 1a displays a schematic representation of the basic principle behind photocatalytic water splitting. Under illumination, photon absorption in a semiconductor induces an electronic transition between the conduction band (CB) and valence band (VB), generating photoexcited charge carriers. After undergoing bulk and surface recombination processes, the remaining photogenerated electrons at the surface reduce protons in water to form H_2_, while the remaining photogenerated holes oxidize water molecules to produce O_2_. The energy band of the semiconductor photocatalyst is important for achieving efficient activity in reduction and oxidation reactions and plays a key role in photocatalytic H_2_ production. The rate of the generation of charge carriers and their separation period are also closely related to the position of the energy band. For water splitting to occur, the semiconductor band edges need to align with the potential levels of water oxidation and reduction, i.e., the photocatalyst should have a valence band maximum (VBM) lower than the water oxidation level, and its conduction band maximum (CBM) should be higher than the hydrogen production level at the same time [18,32]. Therefore, the desirable E_g_ of photocatalyst should be larger than 1.23 eV. 

In a photocatalytic water-splitting system employing TiO_2_, the energy levels of TiO_2_’s CB and VB fulfill the essential energy criteria for triggering the oxidative generation of O_2_ and the reduction process to produce H_2_. The CBM of TiO_2_ determines the reduction power of photogenerated electrons; the higher the CBM level, the stronger the reduction ability of photogenerated electrons. And, the oxidizing power of its photogenerated holes is governed by the VBM; the lower the VBM level, the stronger the oxidation ability of photogenerated holes. As the CBM of TiO_2_ is slightly more negative than the H_2_ evolution level ($E_{H^{+}/H_{2}}$), and the VBM is more positive than the oxygen generation level ($E_{H_{2}O/O_{2}}$), the photogenerated electrons and holes interact with the water molecules adsorbed on the TiO_2_ surface, leading to the reduction of H^+^ into H_2_ and the oxidation of H_2_O into O_2_.

During photocatalysis processes, TiO_2_ photocatalysts are first activated by absorbing photons, which can come from either VIS or UV light irradiation. If the energy of the incoming photon surpasses or meets the E_g_ of TiO_2_, it can stimulate electrons to transition from the VB to the CB, leading to the creation of electron–hole pairs. These pairs consist of negative electrons in the CB and positive holes in the VB. Subsequently, the electron–hole pairs generated via light absorption must be dispersed and moved to the photocatalyst’s reactive surface areas, where they trigger separate reduction and oxidation processes, resulting in the formation of H_2_ (as shown in Equation (1)) and O_2_ (as indicated in Equation (2)).

Photoreduction: 2H^+^ + 2e^−^ → H_2_, E^0^ = 0 V vs. NHE (1)

Photooxidation: 2H_2_O + 4h^+^→ O_2_ + 4H^+^, E^0^ = 1.23 V vs. NHE (2)

Besides the efficient redox ability, photocatalysts should have lower bandgap energy (3.0 eV > E_g_ > 1.23 eV) to be active under VIS light irradiation. Nevertheless, due to its broad bandgap of approximately 3.2 eV, TiO_2_ can only absorb photons with wavelengths of less than 387 nm (primarily UV light), restricting its effectiveness in natural sunlight applications. To extend the absorption range and improve the photocatalytic hydrogen production efficiency, tuning the energy band structure of TiO_2_ with ion doping is an effective method. Ion doping narrows the E_g_ of TiO_2_ and/or induces defective states in the bandgap. Accordingly, photogenerated electron–hole pairs are generated through two electronic excitation mechanisms, which are the band→band transition and the band→bandgap states→band transition, as shown in Figure 1b. For each photogenerated electron–hole pair, the band→band jump requires the absorption of one photon, while the band→bandgap states→band jump requires the absorption of two long-wavelength photons. Clearly, the bandgap states act as a bridge during the generating process of electron–hole pairs with light excitation, and greatly broaden the optical response range. Although the bandgap states can significantly improve the solar absorption efficiency of TiO_2_, some of them are prone to become recombination centers of photogenerated carriers, which inhibits the enhancement of the photocatalytic hydrogen production performance of TiO_2_. The influence of the bandgap states on the photocatalytic hydrogen production efficiency depends on the depth of the defective states in bandgap, as well as the location of the ions forming the bandgap states, which we will discuss in detail later.

Additionally, Figure 1c illustrates that from the initial generation of electron–hole pairs to the catalytic production of hydrogen through water splitting on TiO_2_’s surface, various recombinations occur. Some electron–hole pairs recombine immediately, others during their migration to TiO_2_’s surface, and yet others recombine upon reaching the surface. The surviving charge carriers make it to the surface uncombined and contribute to the water-splitting reactions. Such a process decreases the excited charges by more than 90% [33], according to some scholars, even less than 1% of photoexcited electrons and holes are allowed to participate in the redox reactions forming H_2_ [34]. Therefore, promoting the electron–hole pairs’ separation and transport by modifying TiO_2_ is a very important strategy to improve the efficiency of photocatalytic hydrogen production from water splitting. Specific ions can introduce localized energy states within the bandgap of TiO_2_ or alter its conduction and valence bands. These changes can facilitate the better spatial separation of electrons and holes, reducing their recombination rate. For example, doping with metal ions can create mid-gap states that act as traps for electrons or holes, prolonging their lifetimes and increasing the chances of participating in redox reactions on the surface of the catalyst. Moreover, some dopants can increase the mobility of electrons in the TiO_2_ lattice, leading to a more efficient charge carrier transport. Improved mobility helps in the faster transport of electrons to the surface of the catalyst where they can participate in photocatalytic reactions, thereby reducing the probability of electron–hole recombination.

## 3. Methods of Ion Modification on TiO_2_ for Improved Photocatalytic Property

**Figure 2:**
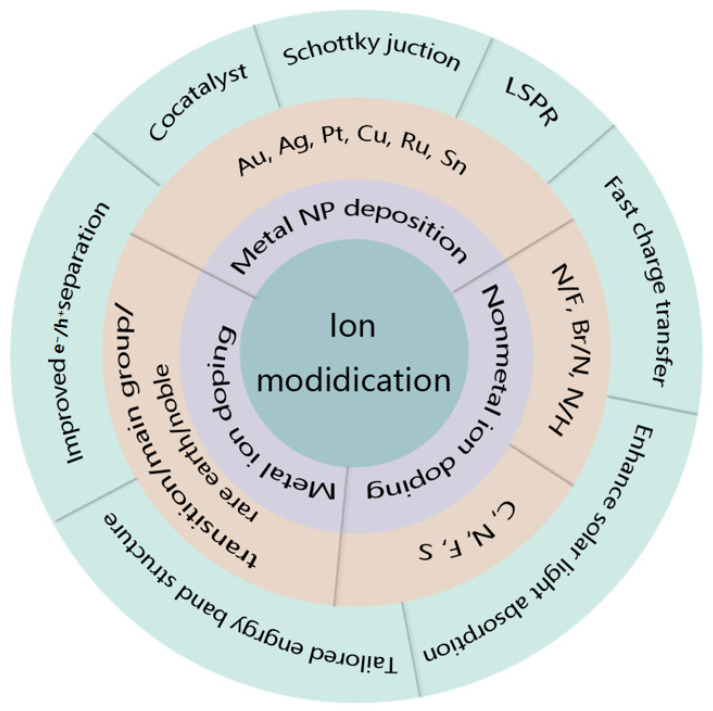
Different strategies employed in the design and development of ion-modified TiO_2_ photocatalysts and the corresponding effects.

For ion-modified TiO_2_, the introduction of dopants changes the nature of chemical bonding, narrows the E_g_ of the material, and/or introduces bandgap states, effectively extending the absorption range into the VIS and even to the near-infrared region. Different ion modification approaches have been documented for enhancing photocatalytic efficiency, including doping hetero atoms to modify the E_g_ [35,36], as well as employing plasmonic metal NPs for light absorption [37]. The properties of dopant elements, such as the ionic radius, electronegativity, and chemical valence of the ions, as well as the dopant concentration, are key factors that affect the modification of the electronic structure of doped materials [38,39,40,41,42,43,44]. It is well known that semiconductors can only absorb photons with energy greater than their E_g_, and the photocatalytic water-splitting reaction only occurs on their surface. Therefore, the properties and concentration of the dopant could determine the range of optical absorption spectroscopy, the dispersal and relocation characteristics of light-induced electron–hole pairs, and the number of active sites on the surface of TiO_2_-based materials. In the following section, as depicted in Figure 2, we discuss the ion modification methods for enhancing the photocatalytic properties of TiO_2_, namely metal doping, metal NP deposition, and nonmetal doping. For each method, we will provide some examples (shown in the secondary outer shell of Figure 2) and discuss the underlying mechanism for the improved photocatalytic efficiency resulting from the effects caused by the ion modification (illustrated in the outermost shell of Figure 2). Moreover, we list the H_2_ generation efficiency, quantum efficiency, durability, and corresponding fabrication methods and test light sources for some typical ion-modified TiO_2_ in Table 1 to present a concise overview of the various modification methods.

### 3.1. Metal Ion Doping

#### 3.1.1. Transition Metal Cations

To optimize the harnessing of solar energy, substantial efforts have been made to enhance the VIS light photocatalytic activity of TiO_2_ by doping it with metal ions [39,53]. The incorporation of transition metal ions reduces the semiconductor’s E_g_ by introducing impurity energy levels (either donor or acceptor levels) into TiO_2_’s band structure, thus activating the photocatalysts in the VIS light spectrum [19,39]. This process can also alter TiO_2_’s crystallinity, potentially creating lattice defects that reduce the recombination of electrons and holes. Furthermore, establishing donor or acceptor levels within the forbidden band of TiO_2_ and generating bandgap states through interaction with the TiO_2_ VB states enhances the spectral response.

A variety of transition metal (such as Ag, Au, Y, Cu, Fe, Pt, Cr, Pd, Zn, Co, and Ni) cation-doped TiO_2_, synthesized with the hydrothermal method, have been widely studied to effectively utilize solar light [39,40,41,45]. Among them, Cu-doped TiO_2_ was selected for further investigation due to its exceptional reactivity. It is crucial to precisely control the doping concentration to achieve optimal results, as excessive dopant levels can serve as recombination centers and degrade photocatalytic efficiency. Thus, determining the appropriate dopant amount for improved charge trapping and separation efficiency is essential. Systematic studies were conducted to optimize the doping concentration and calcination conditions to enhance the photocatalytic activity of Cu-doped TiO_2_. In tests for photocatalytic hydrogen production, Cu-doped TiO_2_ with 0.5 mol% doping, calcined at 650 °C, exhibited superior activity compared to commercial TiO_2_, with hydrogen evolution rates of 200 μmol·$g_{cat}^{-1}$·h^−1^ under UV-A irradiation and 280 μmol·$g_{cat}^{-1}$·h^−1^ under UV-B irradiation. The electronic structure analysis using CASTEP for Cu-doped TiO_2_ revealed that Cu doping introduces states near the valence band edge and reduces the E_g_ [40]. Montoya et al. studied TiO_2_ modified with transition metals (Co, Ni, and Cu) and found that H_2_ evolution rates increased from 0.50 mmol·$g_{cat}^{-1}$·h^−1^ for P25-TiO_2_ to 8.50 mmol·$g_{cat}^{-1}$·h^−1^ for Cu-doped TiO_2_ [46]. Hu et al. prepared Cu-doped TiO_2_ films in varying atmospheric conditions through a straightforward magnetron sputtering technique [45]. The Cu-doped TiO_2_ sample fabricated under an oxygen-rich atmosphere demonstrated high H_2_ production rates of 2.80 μmol·cm^−2^·h^−1^, which is 55 times higher than that of pure TiO_2_. This result is both surprising and encouraging, indicating that significant improvements in photocatalytic performance can be achieved with relatively simple methods. To further explore the impact of Cu doping concentration on TiO_2_’s photocatalytic activity, the electronic structure of Cu-doped anatase TiO_2_ with various Cu concentrations was calculated using first-principle GGA+U calculations. The findings indicate that Cu doping induces a perturbation in the electronic structure, with the most notable feature being the appearance of metal-induced gap states that reduce the E_g_ of the host TiO_2_. The E_g_ decreases with increasing Cu concentration, and both the positions of the VBM and CBM depend on the Cu concentration, suggesting that an optimal Cu concentration exists for achieving high H_2_ generation efficiency [41].

**Figure 3:**
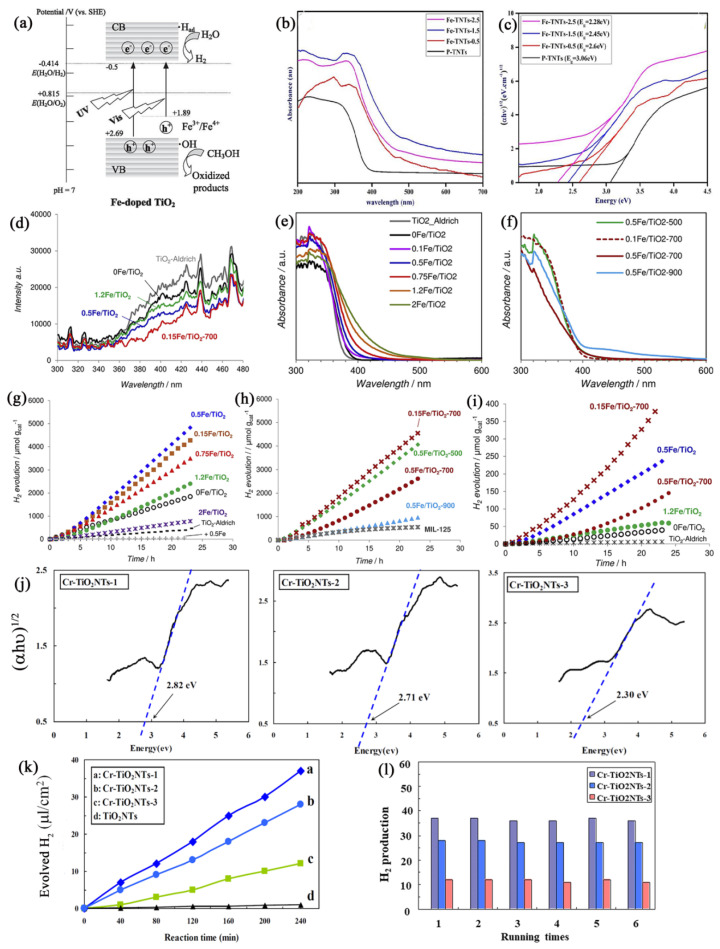
(**a**) Illustrative process for hydrogen generation using Fe-doped TiO_2_ [39]. (**b**) Absorbance spectra and (**c**) Tauc plots of pure and Fe-doped TiO_2_ nanotubes (Fe-TNTs) [42]. (**d**) Photoluminescence and diffuse reflectance spectra for TiO_2_-Aldrich and pure and Fe-doped TiO_2_ synthesized through the one-step (**e**) and two-step (**f**) methods; hydrogen evolution under the UV light irradiation of TiO_2_-Aldrich and the Fe-doped TiO_2_ photocatalysts with the one-step (**g**) and two-step (**h**) method; (**i**) hydrogen evolution under VIS light irradiation using TiO_2_-Aldrich and pure and selected Fe-doped TiO_2_ photocatalysts [43]. (**j**) Bandgap measurements, (**k**) quantitative hydrogen production, and (**l**) hydrogen evolution as a function of running time for various Cr-doped TiO_2_ nanotubes (Cr-TiO_2_ NTs) samples [95].

Fe is frequently chosen as a doping agent in transition metals due to its advantageous electronic structure and the similarity in ionic radius between Fe^3+^ (0.64 Å) and Ti^4+^ (0.68 Å). TiO_2_ doped with iron is recognized as a semiconductor material with the ability to absorb VIS light and exhibits high photoactivity on H_2_ production performance [39,42]. Reddy et al. reported an improvement in hydrogen generation, achieving 270 μmol·h^−1^ with Fe^3+^ doped TiO_2_ prepared via the sol–gel method [47]. This increase in photocatalytic performance can be attributed to impurity energy levels introduced by iron within the bandgap of TiO_2_ (as shown in Figure 3a) [39]. Moreover, Fe^3+^ doping can efficiently separate photogenerated charge carriers by trapping photogenerated electrons or capturing photogenerated holes in the defect states [47]. Numerous investigations have examined how the Fe^3+^ concentration affects the photoactivity of iron-doped TiO_2_ nanostructures synthesized through different methods [39,42,43]. Wang and co-researchers observed an enhancement in the photocatalytic performance of TiO_2_ when doped with 0.05 wt% Fe but noted a decline in activity as the Fe concentration was raised to 0.5 and 1.0 wt% [44]. While samples with higher doping levels captured more light, the efficiency of photocatalytic activity was largely influenced by the rate at which photogenerated electron–hole pairs recombined. Thus, it is beneficial to develop a method that allows for increased iron doping without elevating the recombination rate of these pairs. Traditional fabrication techniques like sol–gel synthesis, co-deposition, and hydrothermal methods are popular for creating iron-doped TiO_2_ but they tend to be labor-intensive, often requiring multiple steps. Zhao and colleagues introduced a streamlined microwave–hydrothermal approach to produce TiO_2_ photocatalysts with varying iron doping levels [39]. The UV–VIS diffuse reflectance spectra indicate that increasing iron concentration led to a red shift in absorbance from 400 to 550 nm. With a doping level of 0.5% iron, the E_g_ of TiO_2_ was reduced to 2.59 eV, achieving optimal photoactivity with a hydrogen production rate of 10.95 μmol·h^−1^. These samples, compared to undoped TiO_2_, had smaller crystal sizes and a larger surface area, enhancing both electron–hole separation and electron mobility [39,94]. In addition, the direct calcination method and a two-step method were separately applied to fabricate Fe-doped TiO_2_ nanopowders and Fe^3+^ ions are highly dispersed in the TiO_2_ lattice. The correlations between synthesis conditions and the behavior of iron-doped TiO_2_ (e.g., iron content, phase composition, and particle size) were examined. The UV–VIS absorption spectroscopy is shown in Figure 3e,f; a noteworthy red shift of the absorption edge occurs towards VIS light with increasing the Fe contents (Figure 3e). The absorption spectra of the sample treated at the highest temperature, specifically 0.5 Fe/TiO_2_-900 (Figure 3f), demonstrated a significant decrease in the bandgap, likely due to the rutile phase formation. The photoluminescence emission analysis (Figure 3d) of undoped TiO_2_ and selected Fe-doped TiO_2_ samples with varying iron concentrations (0.15, 0.5, and 1.2 wt% Fe) revealed that lower iron levels effectively decreased the electron–hole pair recombination rate. The photocatalytic efficiency for hydrogen production under UV and VIS light was evaluated using a sacrificial electron donor, as shown in Figure 3g–i. Hydrogen production efficiency increased threefold from 76.0 to 230 μmol·$g_{cat}^{-1}$·h^−1^ for 0.5 Fe/TiO_2_ sample compared to undoped TiO_2_, showing the significant impact of iron incorporation under UV light. The photocatalytic efficiency of Fe-doped TiO_2_ with a dual-phase structure was found to vary with iron content (Figure 3h). The photocatalytic hydrogen production was significantly enhanced in the sample 0.15 Fe/TiO_2_-700, achieving an increase from 132 to 215 μmol·$g_{cat}^{-1}$·h^−1^, outperforming the 0.5 Fe/TiO_2_-700 sample despite having the same TiO_2_ phase ratio. This enhancement is attributed primarily to the concentration of iron dopant. A noteworthy link was identified between the photocatalysts’ particle size and their ideal iron concentration. A similar pattern was noted under VIS light (Figure 3i), where the rate of hydrogen production rose with moderate iron doping levels. Adjusting the pre-carbonization temperature allowed for the control of both the crystal size and the phase composition of the materials. When assessing the photocatalytic performance of Fe-doped TiO_2_ in splitting water to generate hydrogen, the most effective results were observed at iron concentrations of 0.15 and 0.5 wt%, surpassing the efficiency of both pure TiO_2_ and commercial anatase under both VIS and UV light exposure [43]. Recently, Mie et al. reported that the Fe-doped TiO_2_ nanotubes with different contents of Fe were synthesized via a one-step anodization method [42]. All TiO_2_ materials with the doping of Fe displayed a red shift in the absorption edges (Figure 3b), and the E_g_ decreases with Fe doping content increases (Figure 3c). The sample with a doping of 0.5% Fe exhibits the best photocurrent performance. To elucidate the enhancement in VIS light photocatalytic efficiency, density functional theory calculations were conducted on iron-doped titanium dioxide. The findings suggest that iron incorporation generates impurity levels close to the VB, which decreases the E_g_. This contributes to better electron–hole separation, increased electron transfer efficiency, and improved photocatalytic activity for water splitting [39].

The transitional metal Cr-doped TiO_2_ materials were prepared, and their photocatalytic performance was explored with increases in the Cr concentration [95,96]. Since the Cr ionic radius is nearly identical to that of the Ti^4+^, the Cr cation can be conveniently incorporated into the TiO_2_ crystalline network [96]. TiO_2_ nanotubes with 0.02 at% Cr doping were fabricated and exhibit a high degree of anatase crystallinity, which leads to a significant increase in photocurrent in comparison to pure anatase TiO_2_ [97]. However, when the Cr content was increased, defect effects in the crystal dominated, the separation of photoexcited electron–hole pairs was reduced and led to a decreased photocurrent [97]. Momeni reported that the TiO_2_ nanotubes doped with different contents of Cr were fabricated directly using a single-step process [95]. The Cr doping can increase the VIS light absorption of Cr-doped TiO_2_ relative to undoped TiO_2_ nanotubes, and the E_g_ reduces with the increase in Cr content, as shown in Figure 3j. The Cr-doped TiO_2_ nanotube samples exhibit the improvement of the photocatalytic water splitting properties, and the sample (Cr-TiO_2_ NTs-1) with the optimum content of Cr displayed better photocatalytic performance than the undoped TiO_2_ and other Cr-doped samples, as shown in Figure 3k,l. This can be ascribed to the efficient separation of photoexcited electron–hole pairs in the TiO_2_ nanotube with the appropriate Cr amount [95]. 

#### 3.1.2. Main Group Metal Cations

The main group of metal elements has been employed to modify TiO_2_ to enhance the performance of the photocatalytic performance of TiO_2_. Recent studies have shown that Sn^4+^ doping can adjust the crystal phase of TiO_2_, and the charge transfer between Ti^4+^ and Sn^4+^ in the Sn-doped TiO_2_ system reduces charge carrier recombination, which results in increasing the water splitting efficiency for Sn-doped TiO_2_ [53]. Mg doping in anatase TiO_2_ effectively reduces the deep intrinsic defect states and diminishes the shallow ones. Thus, the effective and stable photocatalytic splitting of water using sunlight was achieved with hollow anatase TiO_2_ spheres after they were doped with Mg. The rates of H_2_ and O_2_ evolution can reach up to 850 μmol·$g_{cat}^{-1}$·h^−1^ and 425 μmol·$g_{cat}^{-1}$·h^−1^, respectively. First-principle calculations suggest that the reduction in defect states is primarily due to the distinct electronic structure of the Mg dopant [69]. Al- and Zn-doped TiO_2_ nanotubes were produced using atomic layer deposition, and their photocatalytic activities were investigated [50]. Exceeding the optimal concentration, further Al doping diminished the photocatalytic effectiveness of TiO_2_ as it led to the creation of charge recombination sites and a decrease in hydroxide radicals. In the case of Zn doping, there exists a specific range where Zn addition enhances photocatalytic performance and improves photoelectrochemical efficiency significantly. At a Zn doping ratio of 0.01, the rate of hydrogen production from water splitting was six times greater than that of the commercial P25 TiO_2_. This is because the Fermi level of Zn-doped TiO_2_ was shifted up to provide more electrons to the CB. Furthermore, the presence of Ti^3+^ sites on the surface and surface O vacancies was crucial in enhancing the photocatalytic process [50]. Recently, TiO_2_ NPs were fabricated for water splitting via doping with different levels of Sr at low temperatures [29]. The TiO_2_ materials doped with 1% Sr showed the best photocatalytic water separation performance with a H_2_ production of 26.3 mmol·$g_{cat}^{-1}$. The increase in photocatalytic performances was attributed to the increased specific surface areas and the tuning of the bandgap achieved through Sr doping [29]. Zhang et al. have prepared the mesoporous nanocrystal TiO_2_ with Ru doping through a one-step corroding process and studied the effect of the special morphologies on the photocatalytic property of Ru-doped TiO_2_. The results showed that Ru-doped TiO_2_ with special morphologies exhibits high electrocatalytic activity for hydrogen evolution reactions [98].

#### 3.1.3. Rare Earth (RE) Metal Cations

Ionic radii of RE metals being larger than those of the Ti^4+^ ion leads to their accumulation on the TiO_2_ surface, enhancing the effective surface area of the TiO_2_ photocatalyst. Although, TiO_2_ doped with an RE metal element was usually investigated for the photocatalytic degradation of organic pollutants using UV and VIS light [99]. For example, the rare RE (Er and Pr)-doped TiO_2_ NPs with a narrow E_g_ of 2.63 eV were obtained, and their photocatalytic performance was enhanced [100]. Incorporating lanthanide metal ions into the TiO_2_ lattice facilitates the entrapment of electrons and increases the electron–hole separation in semiconductor photocatalysts. Recently, Ce-doped anatase TiO_2_ nanocrystals for water splitting under VIS light were synthesized using the sol–gel method [101]. The UV–VIS diffusion reflectance spectrum showed that the absorption region spread to about 550 nm, indicating a red shift of approximately 170 nm due to Ce doping. Ce-doped TiO_2_ exhibited a significant anodic photocurrent effect for water splitting under VIS light (λ > 420 nm) in a photoelectrochemical setup with a three-electrode configuration. Additionally, the electronic structure of CeO_2_ and TiO_2_ have been theoretically analyzed using first-principle calculations. The electronic structure of Ce-doped TiO_2_ is characterized by some overlap and hybridization between the occupied and unoccupied Ce 4f states with the O 2p and Ti 3d states, respectively. This hybridization mainly facilitates the VIS light-responsive properties [101]. 

#### 3.1.4. Noble Metal Cations

Noble metal ions like Pt, Pd, Au, Ag, and Ru can extend the light absorption range of TiO_2_ to VIS light by reducing the E_g_ and enhanc the hydrogen evolution reaction (HER) activity of TiO_2_. Metals such as silver improve photocatalytic performance by increasing surface area, and retarding electron–hole recombination [102]. Gogoi et al. prepared Ag-doped TiO_2_ for water splitting to produce hydrogen using chemical reduction and its E_g_ was reduced to 2.5 eV in contrast to that of TiO_2_. In the presence of Na_2_S and Na_2_SO_3_ sacrificial reagents over 1.5 Ag/TiO_2_, the highest H_2_ production rate achieved was 23.5 mmol·$g_{cat}^{-1}$·h^−1^, with a quantum yield of 19% [51]. Melián et al. reported that incorporating Au and Pt metals into TiO_2_ materials improves the hydrogen evolution efficiency, which, respectively, attained a rate of 1118 μmol·h^−1^ for the 1.5 wt% Au photocatalyst and a 2125 μmol·h^−1^ for the 0.27 wt% Pd photocatalyst [52]. The TiO_2_ doped with 1.00 wt.%, 2.50 wt.%, and 5.00 wt.% Pt were prepared, and the mesoporous 2.50 wt.% Pt-doped TiO_2_ displayed the maximum photocatalytic performance for hydrogen generation, which was ascribed to the reduction in the optical bandgap, increase in electron storage, and decrease in electron–hole recombination [54]. To investigate the impact of noble metal (Au, Ag, Pd, and Pt) doping on the hydrogen production performance of TiO_2_, the first-principle calculation method was utilized. The calculated results indicate that noble metals can facilitate electronic transport between the VB and CB. Among these metals, Ag and Au doping are shown to be more thermodynamically stable compared to Pt, Pd, and Ru doping. Ag doping is advantageous for boosting the hydrogen production performance of TiO_2_, as the hydrogenation of Ag doping diminishes the electronic transfer between the Ti-3d state and the O-2p state [103]. Zhang et al. have prepared the mesoporous nanocrystal TiO_2_ with Ru doping using a one-step corroding process and studied the effect of special morphologies on the photocatalytic property of Ru-doped TiO_2_. The results showed that Ru-doped TiO_2_ with special morphologies exhibits high electrocatalytic activity for hydrogen evolution reactions [98]. 

### 3.2. Metal NP Deposition

**Figure 4:**
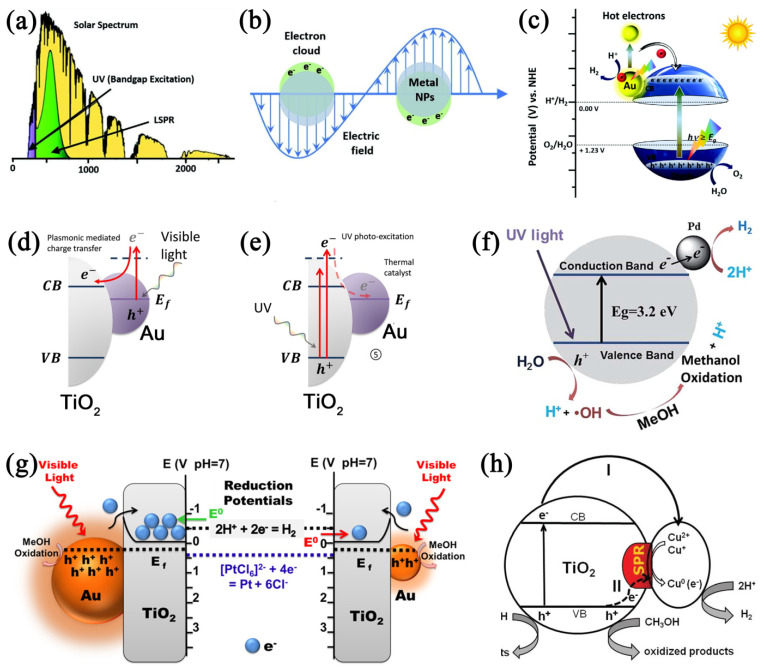
(**a**) Full solar spectrum, UV region for bandgap excitation, and LSPR of metal NP absorption region [112]. (**b**) LSPR of metal NPs, and (**c**) photocatalytic water splitting using plasmonic-mediated TiO_2_ photocatalyst [113]. (**d**) Plasmonic-induced electron transfer from Au deposits to TiO_2_ and (**e**) UV-induced electron excitation in TiO_2_ [112]. (**f**) The synergistic mechanism enhancing H_2_ production through photocatalytic water splitting on Pd/TiO_2_ [72]. (**g**) Diagram of plasmonic hot electron utilization in photocatalysis by adjusting Au NP sizes [114]. (**h**) Suggested pathways for H_2_ generation with Cu-doped TiO_2_ under light above 420 nm (primarily I) and over 500 nm (II) [115].

Although TiO_2_ semiconductors have dominated the field of photocatalysis over the past few decades, the photocatalysts have two main drawbacks, the restricted absorption of photons in the VIS light region and the easy recombination of the photogenerated electron–hole pairs [104]. Various strategies have been suggested to overcome these pivotal shortcomings, including depositing metals on TiO_2_ as co-catalysts to enhance the utilization of photogenerated charge carriers for increased activity [62,65,68,72] or as sensitizers that facilitate electron injection into the CB of TiO_2_ for photocatalytic hydrogen production via the localized surface plasmon resonance (LSPR) effect [67,105]. When metal NPs are coupled with TiO_2_ semiconductors via the metal deposition method, the Schottky junction is formed, and their light absorption range can be extended to VIS and even into the near-infrared region due to LSPR. The improved electron–hole separation efficiency can also be attained by creating Schottky barriers at the semiconductor–metal interface or through the LSPR of noble metals or metal ions. In this case, the incorporated metal serves as an electron trap, capturing electrons from the CB of TiO_2_, which suggests enhanced charge separation efficiency. Surface plasmons are the collective oscillations of free electrons at the surface of conducting material [106], which exhibits strong interactions with light. LSPR excitation (as displayed in Figure 4a,b) happens when the frequency of the incoming light matches the oscillation frequency of surface free electrons against the nuclei’s restoring forces. The photonic energy can be converted into electronic energy by creating hot electron–hole excitation. The production rate of hot electron–hole pairs is 1000 times greater than that of the incident electromagnetic field when the metal NPs are irradiated near its plasmon resonance frequency. As a result, the plasmonic metal NPs locally produce a higher quantity of photoinduced hot charge carriers in TiO_2_ [107,108]. Charge transfer mechanisms at the metal–semiconductor interface include the movement of electrons and holes. The highly energetic hot electrons can be directly injected into the CB of an adjacent TiO_2_ semiconductor. The frequency of the LSPR can be modified by the size, morphology, proximity, and nature of the metal NPs. The LSPR undergoes a wavelength shift towards longer wavelengths as the size increases. Interestingly, recent findings have shown that LSPR in plasmonic NPs facilitates effective light absorption in the VIS and near-infrared spectra [109], leading to the production of hot electrons and holes [110]. While the excitation of LSPR enhances the intense absorption necessary for generating hot charge carriers, the creation of a Schottky junction aids in their separation and transfer [111]. Generally, the metal NPs coupling with TiO_2_ can enhance the photocatalytic activity for water splitting by both increasing the photogenerated electron–hole separation and/or enabling efficient VIS light-harvesting and absorption.

Usually, Ag, Au, and Cu NPs are employed to produce the LSPR effect. It is potentially orchestrated by two mechanisms: direct electron transfer and resonance energy transfer from the metal to TiO_2_ [63]. If the photocatalyst is composed of a physical blend or the metal and TiO_2_ are not directly in contact, a resonance energy transfer mechanism appears. Direct electron transfer occurs when the metal is in direct contact with TiO_2_. For instance, the lifetime of plasmon-excited electrons on Au can increase to 1.5 ns from Au to TiO_2_ when Au and TiO_2_ have direct contact [56]. Under VIS light exposure, hot electrons are photoexcited in Au through LSPR absorption, and then these energetic electrons easily transfer from Au to TiO_2_ (Figure 4c,d) [112,113]. The electron migration between TiO_2_ and metal, whether from TiO_2_ to metal or vice versa, depends on the light irradiation sources. This is because the role of metal NPs, which depends on the light sources, can be different on TiO_2_. Figure 4d,e shows that, under UV light, the TiO_2_ system is activated to generate electrons in CB and holes in VB, and these electrons were captured by metal NPs acting as co-catalysts (Figure 4e). Conversely, under VIS light exposure, they can serve as sensitizers, facilitating the insertion of electrons into the CB of TiO_2_ via LSPR. Therefore, under varying lighting conditions, distinct electron transfer processes are feasible for Au-TiO_2_, i.e., from TiO_2_ to Au under UV light and from Au to TiO_2_ under VIS light [116].

When metal NPs are in direct contact with a TiO_2_ semiconductor, an equilibrium is achieved in the Fermi energy levels, leading to the bending of the semiconductor’s CB, thereby resulting in the formation of a Schottky junction. In the Schottky junction, the work function of the metal NPs exceeds that of the semiconductor. Upon light illumination, the LSPR excitation leads to the production of hot electrons and holes in the plasmonic NPs. Liu et al. conducted density functional theory analyses to verify the charge transfer across the interface between TiO_2_ and Au NPs [31]. Given the n-type nature of the semiconductor, the Fermi energy level appears just below the CB of TiO_2_. Consequently, the formation of a Schottky barrier occurs at the interface due to the downward bending of the CB, aligning the Fermi energy levels of the metal NPs with those of TiO_2_ [117]. The creation of the Schottky barrier at the junction between Au NPs and TiO_2_ permits only high-energy electrons to cross, resulting in the segregation of electrons and holes. Additionally, this rearrangement of charges directly contributes to the formation of an intrinsic electric field at the junction, which inhibits the recombination of charge carriers and improves photocatalytic efficiency. Dosado et al. observed the LSPR effect of Au in Au-TiO_2_ composite, which is utilized for photocatalytic hydrogen production [118]. The Schottky contact formed by Au via the LSPR effect acts as an “electron sink” and decreases the electron–hole recombination, which helps to improve the H_2_ production rate. Also, TiO_2_ and Pd are brought into contact, and the thermal equilibrium and charge redistribution take place, ascribed to the differences in characteristic Fermi levels and the work function of the metal. Pd, having a higher work function than TiO_2_, facilitates the migration of electrons from the semiconductor to the metal where reduction reactions occur, producing hydrogen from H^+^ (as shown in Figure 4f) [72].

According to the classification, metal NPs can serve either as sensitizers or co-catalysts; however, the majority of noble metal NPs typically function in a co-catalytic capacity, capturing excited electrons from TiO_2_. Only a few, such as Ag or Au, can serve as a sensitizer due to the LSPR effect and electron injections into the semiconductor. As co-catalysts, these metal NPs have significantly enhanced the fundamental catalytic efficiency of TiO_2_ photocatalysts [119]. Metal NPs, including Au, Ag, Pt, Cu, Rh, Pd, Ni, Al, and Mg, have been widely utilized as co-catalysts, either by employing their catalytic properties or electron-trapping capabilities [120,121]. To examine the creation and capture of charge when exposed to light with photon energy exceeding the substrate’s E_g_, an innovative analysis method called photoassisted Kelvin probe force microscopy was employed to observe this phenomenon at individual Au NPs. This approach confirmed that the transfer of electrons induced by light from TiO_2_ to the Au NP grows logarithmically with the intensity of light, attributed to the joint effects of electron–hole pair production in the space charge region at the TiO_2_–air interface and at the metal–semiconductor junction [58]. 

#### 3.2.1. Au NPs

The noble Au metal NPs with LSPR combined with TiO_2_ are expected to enhance the photocatalytic activity of catalysts, and the research results in this area are widely reported [57,58,59,116]. The incorporation of Au metal NPs into TiO_2_ has been demonstrated to greatly improve photocatalytic H_2_ production efficiency from water splitting under UV and VIS light. Cronin et al. reported a 66-fold increase in photocatalytic water splitting using Au NP-decorated TiO_2_ under VIS light [122]. This boost in activity is credited to the short minority carrier diffusion length in TiO_2_, with the local electric field near the TiO_2_ surface primarily enhancing the photocatalytic performance, rather than direct charge transfer. The strong local field of surface plasmons in gold NPs effectively enhances the coupling of light with the TiO_2_ surface. Consequently, this leads to a greater separation of electron–hole pairs on the TiO_2_ surface, resulting in an increased rate of photocatalytic hydrogen production [122]. The surface of TiO_2_ was loaded with small-sized Au NPs for improving photocatalytic water splitting under VIS light exposure [61]. The composite containing 0.25 wt% Au NPs on TiO_2_ showed optimal photocatalytic performance at wavelengths of 400 nm and 470 nm, because of the LSPR effect of the Au NPs under VIS light exposure. Brückner et al. described a system of TiO_2_ decorated with plasmonic Au for generating hydrogen under both UV–VIS (320–500 nm) and VIS (400–700 nm) light exposures [123]. The highest rates of hydrogen production, 33 and 2.4 mmol·$g_{cat}^{-1}$·h^−1^, were attained using the Au/anatase–rutile mixture compared to other TiO_2_ phases under UV–VIS and VIS light alone, respectively [123]. Metal NPs with different sizes and shapes have been incorporated with TiO_2_ nanostructures to enhance the absorption of VIS light. This improvement is possible because the LSPR band of the metal NPs can be adjusted based on their size and shape. For instance, the dimensions of plasmonic Au NPs decorated on TiO_2_ significantly affect the photocatalytic production of hydrogen. The efficacy of electron transfer mediated via these plasmons depends on the sizes of the metal NPs, which alter the reduction potentials of the electrons transferred (as shown in Figure 4g) [114]. The influence of particle size on the LSPR of Au NPs was examined, revealing that smaller Au NPs (diameter = 4.4 nm) displayed catalytic activity twenty times greater than that of larger Au NPs (diameter = 67 nm) when coated on TiO_2_ [114]. Recently, Au-TiO_2_ nanosheets were synthesized using Au NPs of various sizes, from approximately 3 nm to 28 nm, to study the influence of NP size on the photocatalytic performance of the catalyst. The findings indicated that smaller Au NPs enhanced the photocatalytic activity on Au-TiO_2_ nanosheets. Specifically, Au-TiO_2_ nanosheets with smaller Au NPs (approximately 3–5 nm) demonstrated the highest rate of hydrogen evolution (around 230 μmol·h^−1^), more than doubling the performance of sheets loaded with larger Au NPs (around 28 nm). In addition, the beneficial components and configuration of the Au-TiO_2_ nanosheets offered extensive surface areas for reactant adsorption, introduced plasmonic effects, and created a Schottky barrier junction. The presence of smaller Au NPs reduced the Schottky barrier height, thus improving the charge separation across the Schottky transfer hub to adjacent TiO_2_ nanosheets [60]. 

#### 3.2.2. Ag NPs

In addition to Au NPs, Ag serves as an excellent metal for exhibiting strong surface plasmon resonance within the preferred wavelength range of 320 to 450 nm. This range aligns closely with the absorption bandgap of TiO_2_ (approximately 3.2 eV or 388 nm), greatly enhancing the separation of photogenerated electrons and holes [51,124]. Thus, it is among the most extensively studied noble metals for developing noble metal-decorated TiO_2_ VIS light photocatalysts [124]. For example, the hydrogen production yield of Ag-TiO_2_ was recorded at 8.1 μmol·cm^−2^ when exposed to combined UV–VIS light, which exceeds the total yields obtained from separate UV (4.2 μmol·cm^−2^) and VIS light (1.6 μmol·cm^−2^) exposures [124]. The hydrogen generation of Ag-TiO_2_ under UV light nearly doubled when subjected to VIS light. This increased efficiency is due to the combined effects of Schottky barrier creation and SPR. Under UV light, a Schottky barrier forms between Ag and TiO_2_, aiding the transfer of photoexcited electrons from TiO_2_ to Ag. Simultaneously, the LSPR effect, triggered by VIS light, promotes the photoexcitation of Ag electrons. This creates a strong local electric field, enhancing the energy of the captured electrons, thereby facilitating the electron transfer and the photoreduction process (H^+^ to H_2_) more effectively [124]. Investigating highly active photocatalysts for hydrogen generation under VIS light, Ag-and-Fe-codoped TiO_2_ NPs were synthesized using an uncomplicated sol–gel approach. The synergistic effect of Ag and Fe reduced the E_g_ of TiO_2_, while the SPR prompted by Ag NPs on the TiO_2_ surface substantially inhibited the photoexcited electrons and decreased the recombination of electron–hole pairs. An optimized dosage of FAT_0.25:2 showed enhanced photocatalytic activity under VIS light exposure compared to pure TiO_2_. Furthermore, after 5 h of VIS light irradiation, Ag-Fe-codoped TiO_2_ nanoparticle FAT_0.25:2 photocatalyst generates 2377.82 μmol·$g_{cat}^{-1}$ of hydrogen from water splitting [21]. The alteration of UV light-responsive TiO_2_ photocatalysts through various plasmonic materials has also been studied to enhance its fundamental properties. L. Sang et al. reported that the bimetallic AgCu combined with TiO_2_ exhibits higher photoelectrochemical activities for water splitting due to the transfer of hot electrons from Ag to Cu and the synergetic effect of diverse LSPRs [66]. The Fe-Ni-codoped and Ag-deposited anatase TiO_2_ (Fe-Ni/Ag/TiO_2_) nanomaterial was successfully synthesized using a straightforward one-pot solvothermal method. Relative to pure TiO_2_, the Fe-Ni/Ag/TiO_2_ composites, with Ag NP sizes ranging from 1.0 to 3.0 nm, exhibited superior optical properties such as enhanced VIS light absorption and enhanced electron–hole pair separation rates. Additionally, the absorption edge of these composites notably shifted to a longer wavelength of 700 nm. Photocatalytic water-splitting experiments were conducted, and the results showed that the composites achieved the highest hydrogen evolution rate, reaching up to 793.86 µmol·$g_{cat}^{-1}$·h^−1^ (λ > 400 nm for 6 h, energy efficiency is 0.25%), significantly surpassing that of pure TiO_2_, which was 9.57 µmol·$g_{cat}^{-1}$·h^−1^ [67]. Qurashi et al. have described a three-component plasmonic photocatalyst Ag/α-Fe_2_O_3_/TiO_2_ for photoelectrochemical hydrogen generation [125]. The Ag NPs were loaded on the surface of α-Fe_2_O_3_/TiO_2_ nanotube arrays. The photoelectrochemical tests indicated that the current density of Ag/α-Fe_2_O_3_/TiO_2_ nanomaterial was fivefold greater than that of bare TiO_2_. In another study, Ag/MoS_2_/TiO_2_-x ternary heterojunction was prepared and the synergistic effect of LSPR, Ti^3+^, and O vacancies were investigated for photocatalytic water splitting. They can not only enhance the absorption of VIS and infrared regions but also effectively inhibit the recombination of electron–hole pairs [126]. It has been noted that Ag’s lower work function relative to TiO_2_ hinders the prevention of hot electron backflow [127]. Consequently, a conduit such as reduced graphene oxide is necessary for facilitating electron movement from Ag to TiO_2_. Utilizing reduced graphene oxide, which serves as an efficient electrical bridge between Ag nanocubes and TiO_2_ nanosheets, the newly developed photocatalyst demonstrates superior photocatalytic hydrogen production compared to the Ag-TiO_2_ composite. This confirms the high efficacy of TiO_2_-based plasmonic photocatalysts in water splitting. By adjusting the size and shape of the nanocomposites, one can create a photocatalytic system that is not only effective but also stable and reusable for practical applications.

#### 3.2.3. Pt NPs

Nobel Pt NP-modified TiO_2_ was prepared, and under UV–VIS light irradiation, its hydrogen generation efficiency (300.63 μmol·$g_{cat}^{-1}$·h^−1^) far exceeded that of Au-TiO_2_ (57.02 μmol·$g_{cat}^{-1}$·h^−1^). The superior efficiency of Pt-TiO_2_ is attributed to the lower overpotential and higher work function of Pt relative to Au. TiO_2_ is readily activated by UV light, leading to the migration of photoexcited electrons from the CB to either Pt or Au. This enhances the charge separation and hydrogen production of Pt-TiO_2_ compared to pure TiO_2_. Under VIS light irradiation (λ > 420 nm), however, the performance reverses: Au-TiO_2_ (7 μmol·$g_{cat}^{-1}$·h^−1^) outperforms Pt-TiO_2_ (2.3 μmol·$g_{cat}^{-1}$·h^−1^), nearly tripling its efficiency. This reversal may be due to the enhanced excitation of TiO_2_ under VIS light through the intense localized electric fields generated with Au’s plasmon excitation, and the transfer of higher energy electrons from excited Au to TiO_2_ [128]. Wang et al. explored the influence of particle size on the performance of Pt-TiO_2_ photocatalysts for HER through density functional theory simulations. Their findings revealed that ultrafine Pt clusters, comprising fewer than two atomic layers, effectively gather photoinduced electrons from the TiO_2_ bulk. This is attributed to their relatively low highest occupied molecular orbital levels when compared to the CBM of TiO_2_. In contrast, larger Pt particles, exceeding two atomic layers, serve as the active site for hydrogen coupling to catalyze HER. Therefore, particles that approximate a two-layer thickness of Pt, about 1 nanometer in size, emerge as the optimal catalysts for photocatalytic HER [129]. The dependency of the hydrogen production activity of the M-TiO_2_ photocatalyst on the metal co-catalyst (M = Au, Pt, Pd) was studied. The investigation revealed that photocatalysts composed of 1 wt.% Pd-TiO_2_ yielded the peak hydrogen generation rates, with co-catalytic performance ranking in the sequence of Pd being superior to Pt, which was roughly equivalent to Au [130]. Single-atomic site catalysts have garnered significant interest for their unparalleled efficiency in atom utilization and superior catalytic performance. For instance, Hejazi et al. found that an optimized single-atom Pt deposition can increase the normalized photocatalytic performance of a sputtered TiO_2_ material by 150 times compared to a conventional Pt NP-deposited TiO_2_ surface [70]. Hu et al. synthesized a single-atomic Pt site photocatalyst using defective TiO_2_ nanomaterial as support for water splitting. Owing to the ability of surface O vacancies on flawed TiO_2_ nanosheets to stabilize singular-atomic Pt sites, the synthesized photocatalyst demonstrated a significant enhancement in H_2_ generation capability. The rate of hydrogen production reached an impressive 13,460.7 μmol·$g_{cat}^{-1}$·h^−1^, marking an increase of approximately 29.0 and 4.7 times compared to that of pristine TiO_2_ nanosheets and Pt nanoparticle TiO_2_, respectively [71]. 

#### 3.2.4. Pd NPs

Pd NPs were deposited on TiO_2_ nanosheets doped with main-group metal ions, resulting in improved photocatalytic hydrogen production. This enhancement is likely due to the increased absorption of UV–VIS light and the facilitated charge transfer and separation within the catalysts. Especially, the Pd/0.2%K^+^-TiO_2_ demonstrates a superior photocatalytic hydrogen generation rate of 76.6 μmol·h^−1^, exceeding the performance of the unmodified Pd-TiO_2_ by over 200% [73]. Metal clusters anchored on supports are recognized for their potential as efficient co-catalysts in heterogeneous photocatalytic systems, attributed to their distinct geometric configurations and specialized reactive properties. By employing a one-step ball milling technique, the photocatalyst with highly dispersed Pd clusters onto TiO_2_ is fabricated. These Pd clusters establish a specialized interface with TiO_2_ support, capable of facilitating the transformation of smaller clusters into larger Pd NPs throughout the hydrogen generation process via photocatalysis. Furthermore, this photocatalyst sustains a consistent performance during prolonged activity, showing stability over an extended period of 100 h of uninterrupted operation [131]. Samples containing increasing amounts of Nb were prepared using Pd as a co-catalyst and a high-surface-area TiO_2_ support. The optimal configuration consisted of 1 mol. % Nb and exhibited a remarkable affinity for harnessing sunlight, with a quantum efficiency value of 2.8%, surpassing that of the bare TiO_2_ reference sample by over 3.3 times [132]. The TiO_2_ supported by a Pd-Cu (Pd/Cu at a ratio of 3:1) co-catalyst demonstrated a quantum efficiency of 2.7%, showing an improvement factor of 1.85 compared to the one with a monometallic Pd co-catalyst. This increase may be attributed to the alloy-creating electron-deficient noble metal centers relative to the Pd reference, and these centers play a key role in altering the chemical properties of the co-catalyst, enhancing hydrogen production [133].

#### 3.2.5. Ru NPs

A series of Ru catalysts supported in TiO_2_ were tested under UV and VIS illumination conditions. The quantum efficiency was attained by measuring the optical performance of the catalysts and the hydrogen photo-production reaction rate. The catalyst demonstrates maximum effectiveness at a concentration of 3 wt.% Ru, achieving a quantum efficiency of approximately 3.0% under UV light and 0.6% under VIS light [48]. In the experiment, Ru atoms are atomically dispersed across multi-edged TiO_2_ spheres to enhance photocatalytic hydrogen production. The data indicate that photogenerated electrons are efficiently transferred to the dispersed Ru atoms for hydrogen production. The multi-edged TiO_2_ structure also supports improved charge separation and transport. The optimized catalyst achieved a hydrogen evolution rate of 7.20 mmol·$g_{cat}^{-1}$·h^−1^, significantly exceeding the performance of Pt-based co-catalyst systems and ranking among the top-reported results [49]. 

#### 3.2.6. Cu NPs

The advancement of affordable and effective co-catalysts plays a pivotal role in enhancing the efficacy of established photocatalysts for hydrogen evolution. While Au and Ag are common choices, Cu stands out as a more abundant and cost-effective alternative. Consequently, the process of depositing Cu on TiO_2_ has garnered significant interest [74,75,134,135]. Sang et al. explored the impact of the size of Cu NPs placed on TiO_2_ nanotube arrays in relation to photocatalytic hydrogen production [76]. They found that Cu NPs with an approximate diameter of 30 nm not only display a potent LSPR effect but also contribute to reducing the recombination rate of charge carriers and the resistance to electron transfer. Sang et al. described the application of an eco-friendly plasmonic Cu-and CuO-modified TiO_2_ material in photoelectrochemical water splitting. Copper NPs manifest a distinctive absorption peak around 550 nm and enhance the photocatalytic activity during water splitting by efficiently facilitating charge carrier separation [78]. They also documented that Cu-TiO_2_ is employed for the photocatalytic [115]. From Figure 4h, it can be seen that the Cu^2+^/Cu^+^ species were reduced to Cu via capturing electrons from the CB of the TiO_2_ sample under VIS light exposure (>420 nm). Additionally, under illumination with light wavelengths exceeding 500 nm, the LSPR phenomenon of Cu begins to exert its influence. The synergistic impacts of both Ag and Cu have been studied on decahedral anatase TiO_2_ particles, which feature eight equivalent (101) facets and a pair of (001) facets, in the context of generating hydrogen when exposed to VIS light [136]. Due to the inclusion of Cu, the formed Schottky barrier is higher than Ag alone, resulting in an accelerated transfer and capture of electrons excited with light. Recently, copper NPs under 5 nanometers were effectively attached to the surface of TiO_2_ through a simple and gentle milling method. The most efficient and persistent hydrogen production is achieved using Cu-TiO_2_ that contains 2.0 wt% Cu and with Cu NP sizes ranging from 2 to 4 nm. This configuration ensures the enhanced separation of photoinduced charges, as evidenced by the high and consistent photocurrent observed [74]. Copper was employed as an alternative to Pt and deposited onto TiO_2_ nanomaterial to improve the efficiency of hydrogen generation via photocatalysis. Investigations revealed that the Cu in its Cu_0_, and not CuO_x_, serves as the catalytically active site driving the reaction forward. The most effective hydrogen production rate for the Cu-modified TiO_2_ was observed at 1023.8 μmol·h^−1^ with a metal loading of 0.1 wt%, which is nearly 20-fold higher than the rate for unmodified TiO_2_ (49.4 μmol·h^−1^) and comparable to the rate achieved with Pt-TiO_2_ (1161.7 μmol·h^−1^). Metal Cu enhanced carrier separation and lowered the hydrogen evolution overpotential, thereby boosting the activity of photocatalytic hydrogen generation [75]. The synthesized TiO_2_ NPs coupled with spherical Cu particles served as the primary photocatalyst and co-catalyst, respectively, for the conversion of solar energy into hydrogen. The incorporation of the Cu particles initiates and enhances the photocatalytic hydrogen generation by facilitating the separation of electron–hole pairs and promoting charge transport [77]. 

#### 3.2.7. Sn/Ni/Co NPs

Metallic Sn NPs, as an Earth-abundant new hydrogen production co-catalyst, have come into efficient contact with TiO_2_ photocatalyst to significantly enhance the HER via a direct photoinduced method. This Sn-based co-catalyst markedly improves the hydrogen production performance of TiO_2_. Specifically, the Sn-TiO_2_ composite with a 3 wt% Sn content achieved the most impressive hydrogen generation rate, registering at 553.1 μmol·$g_{cat}^{-1}$·h^−1^, which is nearly 44-fold greater than that of the unmodified TiO_2_, which produced hydrogen at a rate of 12.6 μmol·$g_{cat}^{-1}$·h^−1^ [82]. Co-catalysts hold significant potential to boost photocatalytic efficiency by expanding the absorption wavelength range through adjustments in the energy band structure, and by enhancing the separation and transportation of photoexcited charges via the creation of type II heterojunctions [137,138,139,140] or nano-twin-induced phase junctions [141]. The atomic Ni co-catalyst on TiO_2_ NPs was prepared via a new molten salt method, which facilitates the atomic dispersion of Ni ions on TiO_2_ with the formation of O vacancies. The synergistic effect of atomic Ni co-catalyst and O defects leads to a fourfold H_2_ production rate compared to the output from a Ni co-catalyst loaded on TiO_2_ synthesized through the impregnation technique [79]. Cu- or Ni-embellished semiconductor materials stand as promising economical substitutes for noble metal-decorated photocatalysts. Bimetallic NiCu NPs display even greater potential, offering a notable improvement in photocatalytic hydrogen production when contrasted with single-metal Ni or Cu systems [81]. MuÇoz-Batista et al. have recently conducted research on phase-contact engineering employing mono- and bimetallic non-noble metal co-catalysts (Cu-Ni) in combination with TiO_2_ for the photocatalytic generation of hydrogen [142]. The findings revealed that, under operational conditions, Cu was more reactive than Ni in the bimetallic system. Furthermore, the photocatalytic activity benefited from a core–shell arrangement, with Cu_0_ at the core and Cu II on the exterior surface [142]. An effective approach was used to develop enhanced photocatalysts for H_2_ production through photocatalysis by replacing noble metals with more readily available elements, serving as co-catalysts for both HER and oxygen reduction. For instance, TiO_2_ photocatalysts modified by Co and Ni co-catalysts were tested with photocatalytic hydrogen production. Hydrogen production over a 6 h period using TiO_2_ enhanced with both Co and Ni (0.1%Co + 0.2%Ni-TiO_2_) roughly doubled (2456 μmol H_2_) compared to TiO_2_ augmented solely with Co (1180 μmol H_2_ for 0.1% Co/TiO_2_) or Ni (1127 μmol H_2_ for 0.2% Ni-TiO_2_). The notable photocatalytic hydrogen generation performance observed with Co- and Ni-decorated TiO_2_ is attributed to the augmented photoactivity and efficient charge carrier separation, a result of the combined effect of Co and Ni, which, respectively, act as active sites for HER and oxidation processes [143]. Moreover, the introduction of non-precious metals such as Ni and Al into TiO_2_ has also shown noteworthy photocatalytic hydrogen production capabilities, attributed to their LSPR effects when exposed to solar illumination [80,144]. 

#### 3.2.8. Transition Metal Nitride/Carbide NPs

Similar to precious metals, nanoscale transition metal nitrides including TiN and ZrN have gained attention for their application as plasmonic systems in the realm of photocatalysis [145,146]. These transition metal nitrides exhibit superior plasmon resonance and offer greater stability—both chemical and thermal—alongside enhanced durability and resistance to corrosion when contrasted with precious metals [147,148]. Nanomaterials made from transition metal nitrides are able to contribute a larger number of energetic electrons to the CB of TiO_2_ than Au NPs. Moreover, the manufacturing costs associated with transition metal nitrides are considerably lower in comparison to Au or Pt NPs. Naldoni et al. described an enhanced wide-spectrum hot electron harvesting for photocatalytic water splitting using TiN plasmonic structures [145]. In comparison to the TiO_2_/Au configuration, the TiO_2_/TiN pairing demonstrated superior photocurrent generation for water splitting. This is attributed to TiN NPs providing extensive light absorption across a 500–1200 nm wavelength range, coupled with the formation of an Ohmic contact between TiN and TiO_2_, which assists in efficient electron collection [145]. Furthermore, the study investigated the potential of two-dimensional titanium carbide MXene, Ti_3_C_2_T_x_ (T_x_ = O, OH, F), as effective co-catalysts for hydrogen generation, utilizing TiO_2_ as the primary photocatalyst. The research found that the hydrogen production rate via photocatalysis using an optimized single-layer Ti_3_C_2_T_x_/TiO_2_ composite exceeded that of pure TiO_2_ by more than 9 times and was 2.5 times greater than that achieved with a multi-layered composite. This significant boost in performance is credited to the exceptional electrical conductivity of the single-layer Ti_3_C_2_T_x_ and the effective separation of charge carriers at the interface between MXene and TiO_2_ [149]. Recently, copper hexacyanocobaltate has been assessed as a promising co-catalyst to couple with standard TiO_2_. The composite demonstrated superior hydrogen production capabilities, outperforming pure TiO_2_ by a factor of up to 16. Moreover, this composite surpassed the efficiency of traditional TiO_2_ modified with copper and cobalt oxides. The improved performance of the TiO_2_/Cu_3_[Co(CN)_6_]_2_ composite is attributed to the effective separation of photoinduced charge carriers and the accelerated electron transfer from the photocatalyst to the reactants [150].

### 3.3. Nonmetal Ion Doping

Doping various transition metals into TiO_2_ is one scheme to improve the VIS light photocatalytic property. However, the doped materials require expensive ion implantation equipment, and such metal doping introduces the localized d-states deep into the bandgap of TiO_2_, which usually serves as the recombination centers for photogenerated carriers, thereby reducing photocatalytic activity. Cationic doping can also cause an unfavorable shift in the CB, pushing it below the redox potential of H_2_O. This shift can render the material inactive for photocatalytic H_2_ production from water splitting. Another approach to developing photocatalysts that are responsive to both UV irradiation (290–400 nm) and VIS light (400–700 nm) is to dope TiO_2_ with anions such as N, C, S, B, F, and P [30,87,88,89,91,151,152,153,154,155,156,157,158,159], which leads to the presence of p states near the VB. For a photocatalyst aimed at overall water splitting, the ideal E_g_ is around 2.0 eV, and the band edges should straddle the redox potential levels of water [160]. The VBM potential of TiO_2_ is significantly lower than the water oxidation level, while its CBM is slightly above the hydrogen reduction level [161]. Hence, the optimal doping strategy involves significantly elevating the VBM while keeping the CBM at its original position. The nonmetal elements, such as N, C, S, and P, just have the higher occupied p orbital energy level than that of O 2p, and the valence band edge of TiO_2_ controlled by O 2p states should be shifted to a higher-energy region by anion element doping.

#### 3.3.1. Nitrogen Anion

**Figure 5:**
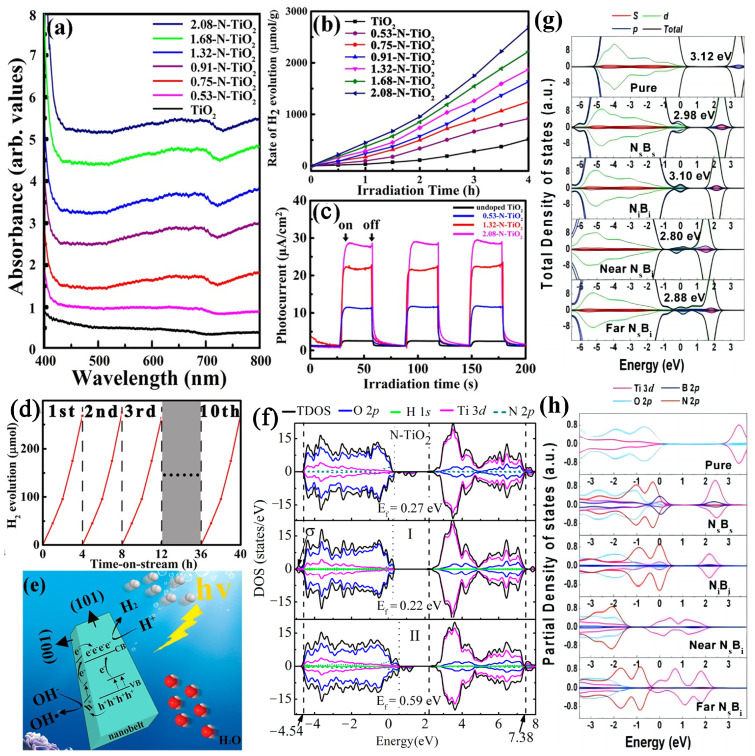
(**a**) UV–VIS light diffuse reflectance spectra, (**b**) H_2_ generation rates, (**c**) cycling stability results for photocatalytic HER, (**d**) photocurrent responses under illumination (λ > 400 nm), and (**e**) diagram of the charge shift process in N-TiO_2_ [84]. (**f**) TDOS and PDOS for pure N-TiO_2_, completely hydrogenated N-TiO_2_ (sample I), and partially hydrogenated N-TiO_2_ (sample II), respectively [169]. (**g**) TDOS of the pure TiO_2_ and the N-B-codoped TiO_2_ under various doping conditions, and (**h**) their corresponding PDOS for Ti 3d, O 2p, N 2p, and B 2p [170].

Nitrogen appears to be a promising dopant due to its similar atomic size to oxygen and low ionization energy. Doping N into TiO_2_ is such an effective approach that could optimize the VBM of TiO_2_ and make the CBM at the same level [162]. Substituting with N was a highly effective way of reducing the E_g_ and inducing bandgap states by mixing N 2p states with O 2p states [162]. In experiments, N-TiO_2_ has been demonstrated to extend the light absorption edge from 380 nm to VIS and potentially provide photocatalytic performance under VIS light exposure [55,163]. Moreover, ultrafine N-TiO_2_ photocatalysts with improved photocatalytic water-splitting capabilities were effectively produced by utilizing PVP as a nitrogen source through a solvothermal approach [83]. In addition, the nanostructure N-TiO_2_ with a rice grain-like morphology was prepared using sol–gel and electrospinning methods. The hydrogen production for N-TiO_2_ is 28 μmol·h^−1^, which is higher than that of 2 μmol·h^−1^ for TiO_2_. The enhancement can be attributed to nitrogen doping and increased surface area [85]. Kwon et al. utilized a simple approach to nitrogen-doped TiO_2_ nanoparticle-based aerogels for activation under VIS light. Balancing dopant concentration and defects enhances the absorption of VIS light and improves electron–hole pair separation efficiency through nitridation. The N-TiO_2_ nanoparticle-based aerogels decorated with Pd NPs exhibited a substantial improvement in VIS light-induced photocatalytic hydrogen generation (3.1 mmol·$g_{cat}^{-1}$·h^−1^) with exceptional stability over 5 days [55]. A nitrogen content of up to 2360.5 at. % was detected when nitrogen plasma alone was utilized to deposit N-TiO_2_ with a plasma-assisted atomic layer deposition system [164]. The type of nitrogen doping could be varied from predominantly interstitial to completely substitutional, as determined with XPS. UV–VIS spectroscopy measurements revealed a shift in the absorption edge from 350 to 520 nm with doping, suggesting a reduction in the bandgap from 3.1 to 1.9 eV [164]. The single-crystalline TiO_2_ nanowire arrays with a substantial content of nitrogen (up to 1.08 atomic %) doping were synthesized via a hydrothermal method. The study discovered that the threshold of incident photon to the current efficiency spectra for N-TiO_2_ samples is approximately 520 nm, corresponding to 2.4 eV. In the UV region, the incident photon to the current efficiency of N-TiO_2_ samples is restored to equal or higher values compared to pure TiO_2_ materials. It maintains a high efficiency of around 18% at 450 nm [153]. Wang et al. report a significant improvement in VIS light photocatalytic property in N-TiO_2_ nanowire arrays through a post-implantation thermal annealing treatment, which can selectively enrich the substitutional N [165]. The substitutional N doping is essential for activating the N-TiO_2_ to gain greatly improved VIS light photoactivity. The significantly enhanced VIS light absorption and more efficient suppression of the rapid recombination of photoexcited charges result in enhanced photoelectrochemical performance. Schmuki et al. synthesized N-TiO_2_ nanotube arrays for hydrogen production through an ion implantation method [166]. Interestingly, the tube layers exposed to a low dose of N exhibited significantly improved activity, while those implanted with higher doses showed virtually no effect. Zhao et al. synthesized the N-doped TiO_2_, which possesses a small particle size of 4.9 nm with better dispersion and reduced E_g_ of 1.6 eV, using a plasma-assisted sol–gel method. The VIS light photocatalytic activity was enhanced due to the small particle size of N-TiO_2_ supplying more surface active sites and high concentrations of surface oxygen [158]. N-TiO_2_ film with preferred (211) orientation, prepared using RF magnetron sputtering, was studied for water splitting. With the increase in exposed (211) facets, the hydrogen generation rates of N-TiO_2_ films increased from 760 μmol·m^−2^·h^−1^ to 4.50 mmol·m^−2^·h^−1^, and the enhanced property of TiO_2_-based photocatalyst was ascribed to the N doping and the preferred orientation of the films [167]. The surface heterojunction constructed in TiO_2_ can promote water molecule decomposition and enhance charge separation capabilities. Sun et al. discovered that the {101} facets of anatase TiO_2_ are advantageous for producing and transferring more reductive electrons to enhance H_2_ generation in the photoreduction reaction, while the {001} facets demonstrate increased photoreactivity to expedite the reaction involving photogenerated holes. As shown in Figure 5, the prepared N-TiO_2_ nanobelts with a surface heterojunction of coexposed (101) and (001) facets showed stronger VIS light absorption (as displayed in Figure 5a), and higher photocatalytic activity with a hydrogen production rate of 670 μmol·$g_{cat}^{-1}$·h^−1^ (as displayed in Figure 5b) under a VIS light due to the charge pairs’ spatial separation (Figure 5e) and the N doping, compared to undoped TiO_2_. Additionally, the result of cycling stability tests (Figure 5d) displays no obvious decrease in H_2_ evolution, demonstrating satisfactory stability. With an increasing N content, the TiO_2_ nanobelts show a higher photocurrent response under VIS light (Figure 5c) [84]. In the experiment, the electronic structure of the N-TiO_2_ was analyzed using X-ray emission spectroscopy and X-ray absorption spectroscopy, offering a unique opportunity to investigate both the highest occupied and lowest unoccupied states in a material with bulk sensitivity [168]. The N doping resulted in the inclusion of p states at the occupied electronic site and a reduction in the population of the lowest unoccupied d-localized orbitals compared to the d-delocalized orbitals near the CB.

#### 3.3.2. Carbon Anion

Carbon doping offers significant potential benefits compared to other nonmetal doping because of several outstanding properties, such as high electron-storage capabilities, excellent charge-transfer properties, and the ease with which to implant into TiO_2_. Plenty of experimental and theoretical studies have concentrated on analyzing the VIS light absorption performance of TiO_2_ with C doping [89,156,171,172]. Carbon doping broadens the edge of absorption light and notably enhances the photocatalytic efficiency of TiO_2_ under VIS light. During carbon doping, the carbon atoms replace O atoms in the TiO_2_ lattice, creating an O-Ti-C bond. This leads to the formation of a hybrid orbital slightly above the VB of TiO_2_, resulting in improved VIS light absorption [104]. The strategic design of C-doped TiO_2_ nanostructures is very significant for obtaining enhanced photocatalytic properties. Shao et al. observed that the enhanced VIS light photocatalytic property of C-doped TiO_2_ nanorods was ascribed to the C dopant, introducing a new impurity energy level above the VB, effectively reducing the E_g_ [173]. The C-doped single-crystal TiO_2_ nanorod has been synthesized, demonstrating low electron resistance and high electrical conductivity due to its orderly crystal structure and natural formation, which facilitate efficient photogenerated electron–hole pair separation and transfer [174]. The high-content carbon doping of TiO_2_, featuring a hierarchical structure and excellent crystallization, was prepared using the exfoliated MXene supernatant at low temperatures [156]. For the hierarchical high-content C-doped TiO_2_, the photocatalytic hydrogen evolution rate was 9.7 times that for the commercial P25 under the same conditions. This is because the carbon doping induced valence band tail states that retarded the recombination of photoexcited electron–hole pairs and broadened the optical absorption range. In order to understand the mechanism of increased water splitting efficiency in the experiment, the ab initio many-body Green function theory was performed to investigate the C-doped TiO_2_. The results showed that the C_2_ dimer formed on the TiO_2_ surface creates a shallow, delocalized occupied Ti 3d state just below the CBM. This results in a reduced E_g_ due to simultaneous shifts in both the VB and CB, differing from the commonly accepted notion that anionic dopants only influence the VB of TiO_2_ [175]. Shao et al. synthesized N- and C-doped TiO_2_ nanoparticles via hydrothermal and heat treatment processes using chitosan as a natural source of C and N [176]. The doping with C and N reduced the E_g_ and formed a sub-band gap above the VB of TiO_2_, enhancing the composite’s response to VIS light and increasing its photocatalytic property under such conditions.

#### 3.3.3. S/F Anion

Porous anatase TiO_2_ nanopillars doped with S are synthesized using a simple one-step thermal protection technique. When these S-doped TiO_2_ specimens are calcined at 700 °C, they display superior photocatalytic activity under VIS light, achieving hydrogen evolution rates of 163.9 μmol·$g_{cat}^{-1}$·h^−1^. This enhanced performance can be credited to the incorporation of S, their porous configuration, and their high anatase crystallinity [88]. While S doping results in a comparable reduction in the bandgap, doping it into the TiO_2_ crystal structure is challenging due to its large ionic radius. This difficulty is reflected in the significantly higher formation energy needed for S substitution compared to N [152]. Recently, TiO_2_ doped with F elements has attracted much research interest because it exhibited an improved photocatalytic activity under both UV and VIS light. Zhang et al. successfully synthesized the F doping of TiO_2_ nanosheets, which showed dramatically improved hydrogen evolution efficiency. This enhancement is due to their VIS light absorption and rapid charge carrier transfer [177]. Further, selective etching and doping of F^−^ on the (001) facets of anatase TiO_2_ nanosheets were synthesized using TiO_2_ nanosheets that exposed both (001) and (101) facets as precursors. The prepared nanosheet samples were able to significantly enhance the separation of photogenerated charge carriers by reducing the hole transfer pathway and inducing Ti^3+^ and O vacancies into the (001) facets. Consequently, the specimen exhibited outstanding photocatalytic activity under VIS light, achieving a maximum photocatalytic hydrogen production rate of 18.27 mmol·$g_{cat}^{-1}$·h^−1^ and a quantum efficiency of 21.6% at a wavelength of 420 nm [90]. 

#### 3.3.4. N-B/N-H/N-F/C-N/Br-N Codoping

Although the mono-doped TiO_2_ shows some response to VIS light, its absorption of VIS light and photocatalytic efficiency are relatively low. This is because the high formation energy required for high-content anion incorporation into TiO_2_ and the substitutional N doping concentration is remarkably low, which restricts the elevation of VBM [178]. More than one ion doping (codoping)-modified TiO_2_ is considered to be an effective strategy to further enhance its photocatalytic efficiency and has received much attention both theoretically and experimentally [169,179,180,181,182]. The N-B-codoped TiO_2_ showed enhanced photocatalytic performance under UV and VIS light exposures, likely because of a synergistic effect [183,184]. The N-doped TiO_2_ with H incorporation exhibits improved photocatalytic performance under VIS light [185], and the N doping content can be improved from 2% to 4.4% when the N- TiO_2_ was fabricated under NH_3_ atmosphere [186]. To explain the origin of enhanced photocatalytic activity, our group has employed first-principle calculation to systematically investigate N-doped TiO_2_ with H incorporation. The calculated results show that both full and partial hydrogenation could stabilize N-TiO_2_ by greatly decreasing the formation energy of N doping under Ti-rich conditions. It was found that, in comparison with N-TiO_2_, only the partially hydrogenated N-TiO_2_ was responsible for the improvement in the photocatalytic activity because its VBM is shifted upwards by 0.32 eV and the VB states intermix with the broad bandgap states (Figure 5f), leading to enhanced light absorption and charge carrier separation [169]. Lately, the experimental and theoretical researchers also studied N-F-codoped TiO_2_ and found that the codoped materials exhibited high photocatalytic activity under VIS light irradiation [180,187]. Regarding N-B-codoped TiO_2_, as illustrated in Figure 5g, the N_s_B_i_ (substitutional N, interstitial B) variant of codoped TiO_2_ generates substantial mid-gap states when the separation between N and B atoms is large. In contrast, the N_i_B_i_ (interstitial N and B) and N_s_B_s_ (substitutional N and B) variants of codoped TiO_2_ tend to produce localized p states ranging from 0.3 to 1.2 eV above the VBM. Additionally, the optical band edges of these three codoping systems shift slightly towards the VIS spectrum, yet only the N_s_B_i_-codoped TiO_2_ with wider N and B separation distinctly manifests an optical transition. These findings suggest that N_s_B_i_-codoped TiO_2_ predominantly enhances the optical absorption in N-B-codoped TiO_2_ systems. As depicted in Figure 5h, across these three systems, the N 2p states play a crucial role in the doping effects, with the Ti atom effectively collaborating with the neighboring doped atom [170]. Furthermore, the prepared C-N-codoped TiO_2_ has the capacity to extend the optical absorption region into the VIS light range [92,188]. In addition, the hierarchical honeycomb Br-N-codoped anatase TiO_2_ nanosheets were prepared, and they own the enhanced VIS light photocatalytic H_2_ production [93].

## 4. Conclusions and Perspectives

In conclusion, ion-modified TiO_2_ photocatalysts show enhanced photocatalytic H_2_ production from photocatalytic/photoelectrochemical water splitting and are systematically reviewed. The innovations in synthesis methods, the performance of the photocatalytic/photoelectrochemical water splitting of ion-modified TiO_2_, and the proposed mechanisms of the enhanced photocatalytic activity are illustrated. Firstly, for effective hydrogen generation through photocatalytic/photoelectrochemical water splitting, doping TiO_2_ with metals is a viable approach. The possible mechanism of enhanced VIS light photocatalytic activity was that metal doping facilitates the creation of localized states within the bandgap of TiO_2_, which contribute to electronic transitions in the VIS spectrum. Secondly, metal NP-modified TiO_2_ exhibits excellent photocatalytic performance. The metal NPs, acting as photosensitizers and co-catalysts, have the capacity for strong VIS light absorption and facilitate the separation and transfer of photogenerated electron–hole pairs. Lastly, nonmetal doping represents another effective method to enhance VIS light activity and improve the photogenerated carrier separation/transfer efficiency in TiO_2_. The nonmetal doped TiO_2_ for photocatalytic water splitting has been reviewed from various perspectives. The potential sources of VIS light absorption and subsequent photocatalytic processes are discussed. Nonmetal doping reduces the E_g_ and induces localized states within TiO_2_’s bandgap, aiding its VIS light absorption. Doping states such as C 2p, N 2p, and S 3p may be positioned above the VB of TiO_2_ or blended with O 2p to raise the VB. Additionally, defect states such as O vacancies and Ti^3+^ from nonmetal doping could exist above the VB and below the CB of TiO_2_, respectively. The anion codoping could make up for the deficiency of single-anion doping, such as the high formation energy of single-anion doping, thereby further raising the photocatalytic efficiency.

**Figure 6:**
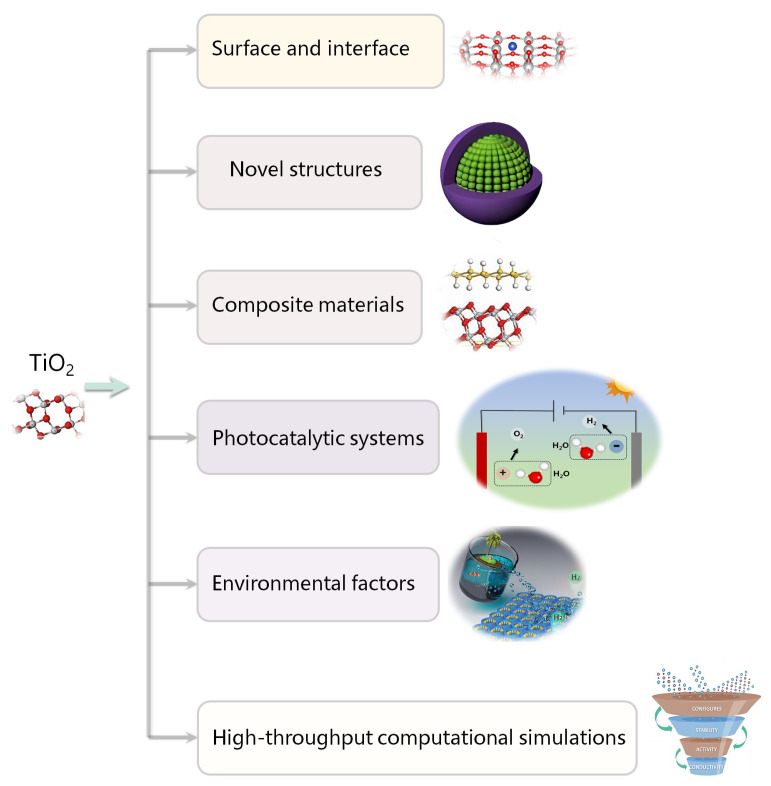
The future directions for ion-doped TiO_2_ photocatalysts for water splitting and hydrogen production.

Although the ion-modified TiO_2_ has played an important role in photocatalysis for both economical and environmentally friendly H_2_ production, there still are many challenges in this field. As shown in Figure 6, the future directions for ion-doped TiO_2_ photocatalysts for water splitting and hydrogen production include the following:

(1) **Surface and interface engineering**. Using ion doping to alter the surface properties of TiO_2_ can optimize interactions with water and enhance catalytic activity at the reaction interface. Studying the interfacial effects between TiO_2_ and doping ions can further increase photocatalytic efficiency.

(2) **Design and synthesis of novel structures**. Developing specific ion-doped TiO_2_ nanostructures, such as core–shell or porous structures, can improve light absorption efficiency and surface reactivity. These structures provide more active sites and higher photon utilization.

(3) **Preparation of composite materials**. Combining ion-doped TiO_2_ with other semiconductors, metals, or carbon-based materials can form composite photocatalysts. These composites can leverage synergistic effects between components to further enhance hydrogen production efficiency and stability.

(4) **Integration and optimization of photocatalytic systems**. Researching and optimizing the entire photocatalytic system, including choices of light sources and reactor design, can ensure the maximum performance of ion-doped TiO_2_ catalysts in practical applications.

(5) **Environmental factors consideration**. Assessing the performance of ion-doped TiO_2_ photocatalysts under different environmental conditions (such as pH, temperature, etc.) and their long-term stability and sustainability can ensure effectiveness and environmental friendliness in real-world applications.

(6) **Application of high-throughput computational simulations**. High-throughput computational simulations offer a promising approach for the development of ion-doped TiO_2_ photocatalysts for water splitting and hydrogen production. The application prospects of this technology in the field are broad and impactful, primarily due to its ability to efficiently screen and predict the performance of a vast array of materials under different conditions. The integration of high-throughput computational results with experimental data can lead to a more comprehensive understanding of photocatalytic mechanisms, validate simulation predictions, and further refine computational models for better accuracy in future predictions.

These research and development directions could lead to more efficient and widely applicable ion-doped TiO_2_ photocatalysts in the field of hydrogen production through water splitting.

## Figures and Tables

**Table 1:** Summary of some ion-modified TiO_2_ samples for photocatalytic hydrogen production performance.

Type	Sample	Fabrication Method	Light Source	H_2_ Generation Efficiency	Quantum Efficiency	Durability, h (Retention, %)	Ref.
Metal ion doping	Cu-TiO_2_	Magnetron sputtering	300 W Xe lamp	2.80 μmol·cm^−2^·h^−1^	--	--	[45]
	Cu-TiO_2_	Hydrothermal	UV-B	280 μmol·$g_{cat}^{-1}$·h^−1^	--	--	[40]
	Cu-TiO_2_ (P25)	Photoassisted deposition	450 W Hg lamp	8.47 mmol·$g_{cat}^{-1}$·h^−1^	7.0%	3 (stable)	[46]
	Co-TiO_2_	Photoassisted deposition	450 W Hg lamp	2.48 mmol·$g_{cat}^{-1}$·h^−1^	2.1%	3 (50–70%)	[46]
	Ni-TiO_2_ (P25)	Photoassisted deposition	450 W Hg lamp	3.39 mmol·$g_{cat}^{-1}$·h^−1^	2.8%	3 (50–70%)	[46]
	Fe-TiO_2_	Sol–gel	Solar light	270 μmol·h^−1^	--	--	[47]
	Fe-TiO_2_	Microwave–hydrothermal	Xe lamp (350–780 nm)	10.95 μmol·h^−1^	--	--	[39]
	Fe-TiO_2_	Impregnation procedure	500 W Xe/Hg lamp (UV light)	230 μmol·$g_{cat}^{-1}$·h^−1^	--	--	[43]
	Ru-TiO_2_	Micro-emulsion	500 W Xe lamp (280–400 nm)	≈4.60 mmol·$g_{cat}^{-1}$·h^−1^	3.1%	--	[48]
	Ru-TiO_2_	Micro-emulsion	500 W Xe lamp (420–680 nm)	≈0.80 mmol·$g_{cat}^{-1}$·h^−1^	0.6%	--	[48]
	ME-TiO_2_@Ru	Sol–gel	300 W Xe lamp	7.2 mmol·$g_{cat}^{-1}$·h^−1^	--	5 (stable)	[49]
	Zn-TiO_2_	Atomic layer deposition	150 W Xe lamp	2.66 mmol·$g_{cat}^{-1}$·h^−1^	4.88%	--	[50]
	Sr-TiO_2_	Hydrothermal	200 W Hg−Xe lamp	3.3 mmol·$g_{cat}^{-1}$·h^−1^	--	24 (98.6%)	[29]
	Ag-TiO_2_	Chemical reduction	UV Lamp	23.5 mmol·$g_{cat}^{-1}$·h^−1^	19%	18 (stable)	[51]
	Au-TiO_2_	Photodeposition	UV Lamp (300–400 nm)	1.118 mmol·h^−1^	--	8 (stable)	[52]
	Pt-TiO_2_	Photodeposition	UV Lamp (300–400 nm)	2.125 mmol·h^−1^	--	8 (stable)	[52]
	Pt/Sn-TiO_2_	Hydrothermal	350 W Xe lamp	39.4 mmol·$g_{cat}^{-1}$·h^−1^	--	9 (stable)	[53]
	Pt-TiO_2_	Sol–gel	15 W Black-Blue lamp (320–410 nm)	0.117 μmol·cm^−3^·h^−1^	22.6%	50 (stable)	[54]
	Pd/N-TiO_2_	Chemical vapor deposition	White LED (400–800 nm)	6.3 mmol·$g_{cat}^{-1}$·h^−1^	5.6%	120 (stable)	[55]
Metal NP deposition	Au-TiO_2_	Sol–gel	300 W Xe lamp (λ > 420 nm)	7.00 μmol·$g_{cat}^{-1}$·h^−1^	--	24 (stable)	[56]
	Au/TiO_2_	Chemical reduction	UV LED (375 nm)	6.661 mmol·gcat−1·h^−1^	1.03%	18 (stable)	[57]
	Au/TiO_2_	Magnetron-sputtering	UV light	1.95 mmol·h^−1^	--	--	[58]
	Au/TiO_2_	Photodeposition	UV-VIS light	360 μmol·$g_{cat}^{-1}$·h^−1^	61.2%	15 (80%)	[59]
	Au/TiO_2_	Urea reduction	350 W Xe lamp	≈230 μmol·h^−1^	15.94%	--	[60]
	Au-P25	Standard Sol	300 W Xe lamp (400 nm)	1.05 mmol·$g_{cat}^{-1}$·h^−1^	--	24 (stable)	[61]
	Au@TiO_2_	Hydrothermal	300 W Xe lamp	4.92 mmol·$g_{cat}^{-1}$·h^−1^	--	18 (stable)	[62]
	Ag/Au-TO_2_	Photodeposition	AM 1.5	718 μmol·$g_{cat}^{-1}$·h^−1^	3.3%	24 (stable)	[63]
	Ag/TiO_2_	Microwave-assisted chemical reduction	16 W Hg lamp (UV), 500 W Xe lamp (VIS)	2.7 µmol·cm^−2^·h^−1^	--	5 (stable)	[64]
	Ag/H-TiO_2_	Pulse electrodeposition	300 W Xe lamp (λ > 420 nm)	124.4 µmol·cm^−2^·h^−1^	--	--	[65]
	AgCu/TiO_2_	Electrodeposition process	AM 1.5 G	246.77 μL·cm^−2^·h^−1^	--	--	[66]
	Ag-Fe/ TiO_2_	Sol–gel	300 W Xe lamp (VIS light)	475.56 μmol·$g_{cat}^{-1}$·h^−1^	--	--	[21]
	Fe-Ni/Ag/TiO_2_	Solvothermal	500 W Xe lamp (λ > 400 nm)	793.86 μmol·$g_{cat}^{-1}$·h^−1^	--	30 (stable)	[67]
	Pt/N-TiO	Sol–gel	400 W Hg lamp (VIS)	772 μmol·$g_{cat}^{-1}$·h^−1^	--	70 (90.67%)	[68]
	Pt/Mg-TiO_2_	Hydrothermal	300 W Xe lamp	850 μmol·$g_{cat}^{-1}$·h^−1^	19.4%	--	[69]
	Pt-SA/TiO_2_	Magnetron sputtering	50 mW laser (325 nm)	≈380 μmol·$g_{cat}^{-1}$·h−1	--	144 (80%)	[70]
	Pt SA/Def-s-TiO_2_	Deposition–precipitation	300 W Xe lamp	13.4607 mmol·$g_{cat}^{-1}$·h^−1^		10 (97%)	[71]
	Pd-TiO_2_	Chemical reduction	300 W Xe lamp (solar simulator)	3.096 mmol·$g_{cat}^{-1}$·h^−1^	3.4%	6 (stable)	[72]
	Pd/K+-TiO_2_	Hydrothermal	300 Xe lamp (UV-VIS light)	76.6 μmol·h^−1^	3.0%	21 (72.1%)	[73]
	Cu-TiO_2_	Ball milling	300 W Xe lamp	9.5 mmol·$g_{cat}^{-1}$·h^−1^	--	20 (73%)	[74]
	Cu-TiO_2_	Situ photodeposition	300 W Xe lamp(λ > 300 nm)	1.0238 mmol·h^−1^	--	36 (stable)	[75]
	Cu/TiO_2_	Electrochemical deposition	Solar simulator (λ > 400 nm)	159.59 μL·cm^−2^·^h^−1^^	--	2 (stable)	[76]
	Cu-TiO_2_	Hydrothermal	300 W Xe lamp	5.566 mmol·$g_{cat}^{-1}$·h^−1^	--	10 (stable)	[77]
	Eosin Y/Cu-CuO/TiO_2_	Two-step electrochemical	Solar simulator	≈118 μL·cm^−2^·h^−1^	--	3 (stable)	[78]
	Ni-TiO_2_	Molten salt	300 W Xe lamp	1.89 mmol·$g_{cat}^{-1}$·h^−1^	--	20 (stable)	[79]
	Ni-TiO_2_ @CMK-8	Solvothermal	300 W Xe lamp	592.67 μmol·$g_{cat}^{-1}$·h^−1^	37.9%	--	[80]
	NiCu-TiO_2_	Electrochemical deposition; Plasma sputtering	LED UV light	7.4 μL·cm^−2^·h^−1^	--	--	[81]
	Sn/TiO_2_	Photoinduced deposition	3 W UV lamp (365 nm)	553.1 μmol·$g_{cat}^{-1}$·h^−1^	1.48%	10 (stable)	[82]
Nonmetal ion doping	N-TiO_2_	Hydrothermal	300 W Xe lamp	323 μmol·$g_{cat}^{-1}$·h^−1^	--	18 (stable)	[83]
	N-doped TiO_2_	Hydrothermal	300 W Xe lamp (VIS light)	0.67 mmol·$g_{cat}^{-1}$·h^−1^	--	40 (stable)	[84]
	N-TiO_2_	Sol–gel and electrospinning	150 W Xe lamp (VIS light)	28 μmol ·h^−1^	--	--	[85]
	N-TiO_2_	RF magnetron sputtering deposition	300 W Xe lamp	4.50 mmol·cm^−2^·h^−1^	--	--	[86]
	N-TiO_2_ with VO	Solvothermal	Solar simulator (λ > 300 nm)	1.035 mmol·$g_{cat}^{-1}$·h^−1^	16%	6 (stable)	[87]
	S-TiO_2_	Thermal protection	VIS light	163.9 μmol·$g_{cat}^{-1}$·h^−1^	--	--	[88]
	TiC@C-TiO_2_	Situ thermal growth	300 W Xe lamp (λ > 400 nm)	558.46 μmol·$g_{cat}^{-1}$	--	6 (stable)	[89]
	F-TiO_2_	Hydrothermal	VIS light	18.27 mmol·$g_{cat}^{-1}$·h^−1^	21.6%	40 (stable)	[90]
	N/F-TiO_2_	Calcination	300 W Xe lamp (λ > 420 nm)	≈11.5 μmol·h^−1^	--	24 (stable)	[91]
	C/N self-doped TiO_2_	Hydrothermal	350 W Xe lamp	332.3 μmol·h^−1^	--	--	[92]
	Br/N-TiO_2_	Hydrothermal	300 W Xe lamp (λ > 420 nm)	2.247 mmol·$g_{cat}^{-1}$·h^−1^	--	--	[93]

“stable” means that the hydrogen production rate does not show significant changes. “--” means that the data were not found in the literature.
